# Does living liver donors’ underestimation about surgical outcomes impact on their health-related quality of life after donation?: a descriptive cross-sectional study

**DOI:** 10.1186/s12955-022-02055-0

**Published:** 2022-10-24

**Authors:** Ye Sol Lee, Chin Kang Koh, Nam-Joon Yi, Kyung-Suk Suh, Kwang-Woong Lee

**Affiliations:** 1 College of Nursing, Seoul National University, 103 Daehak-ro, Jongno-gu, 03080 Seoul, Republic of Korea; 2 College of Nursing, The Research Institute of Nursing Science, Seoul National University, 103 Daehak-ro, Jongno-gu, 03080 Seoul, Republic of Korea; 3 Department of Surgery, College of Medicine, Seoul National University, 101 Daehak-ro, Jongno-gu, 03080 Seoul, Republic of Korea

**Keywords:** Quality of life, Liver transplantation, Living liver donor, Unmet expectations, Informed consent, Patient education

## Abstract

**Background:**

In South Korea, the number of living-donor liver transplantations in 2019 was 1,188. Living liver donors (LLDs) undergo surgery and the postoperative recovery process for altruistic purposes. This study explored LLDs’ unmet expectations about surgical outcomes and examined their impact on the donors’ health-related quality of life (HRQOL).

**Methods:**

This descriptive cross-sectional study utilized a self-reported survey. Data were collected at a university hospital in Seoul, South Korea. Among the 535 LLDs who underwent surgery for donation between January 2011 and March 2021, 124 participated in this study. The Korean version of the 12-item Short Form Health Survey version 2 (SF-12v2) was used to measure the HRQOL of LLDs. Unmet expectations regarding surgical outcomes were measured using four items: pain, length of hospital stay, speed of recovery, and complications. Logistic regression model was applied to determine whether the unmet expectations influence HRQOL in LLDs. Odds ratios with 95% confidence interval were used.

**Results:**

The percentage of the participants who reported that their actual experiences for pain, speed of recovery, hospital stay, and complications were worse than expected were 34.7%, 22.6%, 9.7%, and 7.3%, respectively. Unmet expectations about surgical outcomes were significantly associated with physical and mental HRQOL after controlling for age, sex, education level, income, postoperative complications, recipients’ death, time since donation, and satisfaction with the decision to donate.

**Conclusion:**

LLDs should be supported in obtaining more accurate and realistic information about surgical outcomes to decrease unmet expectations, which may help improve their quality of life.

**Supplementary Information:**

The online version contains supplementary material available at 10.1186/s12955-022-02055-0.

## Background

Due to the cadaveric organ shortage and the availability of advanced surgical techniques in South Korea, 75.2% of liver transplantations conducted in 2019 were living-donor liver transplantations (LDLTs) [1]. The number of living liver donors (LLDs) has increased over the last decade, from 717 to 2008 to 1,188 in 2019 [1, 2]. LLDs in South Korea were mainly immediate family members of the recipients: as of 2019, the donor was a son or daughter (68.0%), spouse (11.3%), sibling (8.9%), parent (3.7%), collateral blood relative such as a nephew or cousin (3.5%), relative by marriage (3.5%), or an unrelated person like a friend (1.1%) [1].

LLDs experience major surgery as a part of the transplantation process. Thus, they take risks for altruistic purposes. The mortality rate for LLDs is 0.2%, and the median morbidity rate is 16% [3]. In South Korea, a study of 245 cases at one university hospital showed a complication rate of 46.1% [4]. Moreover, the donations made by LLDs may affect their later daily lives. Although the levels of health-related quality of life in LLDs are not different or even higher than those in the general population [5–8], donation could be a factor that affects later health-related quality of life (HRQOL). Hesimov et al. [9] revealed that the physical aspects of the quality of life in LLDs decreased immediately after surgery and recovered over the first year. Moreover, demographic factors such as age, sex, education level and donation-related factors such as financial costs, postoperative complications, recipients’ death, time since donation, and the donors’ satisfaction with donation have been reported to be associated with HRQOL [6, 10–14].

Prior to surgery, patients develop their own perceptions and expectations regarding surgical outcomes such as pain, speed of recovery, and side effects, and these perceptions may influence the patient’s postoperative psychosocial outcomes [15]. Developing unreasonably optimistic expectations about surgical outcomes may negatively influence patients’ postoperative experience [15]. Patients’ unmet expectations about surgery have been reported to be negatively associated with postoperative functional improvement [16]. However, the expectations of LLDs regarding surgical outcomes and their relationships with post-donation health outcomes have not been adequately evaluated. Therefore, this study explored whether the expectations of LLDs regarding surgical outcomes were met and determined their relationship with the post-donation HRQOL of LLDs.

## Methods

### Study design

This cross-sectional descriptive study was conducted using a self-report survey method.

### Study participants

This study was conducted at a large tertiary university hospital in Seoul, South Korea. Eligible participants in this study were LLDs aged between 19 years to 64 years within second-degree of kinship with the recipients, who underwent partial hepatectomy between January 2011 and March 2021. Donors who had undergone surgery less than one month previously were excluded. Among a total of 535 potential participants, 124 donors participated in the study.

Data were collected using a self-reported survey and retrospective medical record reviews conducted between February and July 2021. The survey was conducted using web-based or paper forms at outpatient clinics. This study was approved by the institutional review board of the hospital where the data collection was performed. Informed consent was obtained for the survey and the use of clinical data.

## Measures

### Health-related quality of life

A 12-item Short Form Health Survey version 2 (SF-12v2) [17] was utilized to evaluate donors’ HRQOL. The SF-12v2 consists of eight health domains: physical functioning (PF), role physical (RP), bodily pain (BP), general health (GH), mental health (MH), role emotional (RE), social functioning (SF), and vitality (VT). The scores for these scales were aggregated into physical and mental component summary (PCS and MCS) measures. Each item was assessed using a 3-point or 5-point Likert scale, and higher PCS and MCS scores indicated better HRQOL. The SF-12v2 has demonstrated desirable reliability (Cronbach’s α = 0.88) and construct validity in the general Korean population [18]. In this study, the Cronbach’s α was 0.77, and poor HRQOL was defined as more than 0.5 SD below the normative mean of the general Korean population [19].

### Unmet expectations about surgical outcomes

To determine whether the expectations of LLDs regarding surgical outcomes were met, the donors were asked to respond to this retrospective question: How was your actual postoperative experience compared to your expectations prior to the donation? Surgical outcomes were evaluated using four items: length of hospital stay, speed of recovery, pain, and complications. The answer choices were “better than expected,” “as expected,” and “worse than expected.” When the reality was worse than their expectation, it was identified as an unmet expectation. This question was developed for this study on the basis of previous literature [7, 15, 20, 21] and reviewed by five healthcare professionals (a surgeon and four nurses involved in liver transplant). Face validity was assessed through five LLDs.

### Control variables

We collected donors’ sociodemographic and donor-specific information, including age, sex, education level, monthly income, postoperative complications, recipient death, time since donation, and satisfaction with the decision to donate. Postoperative complications were categorized into grades I-IV according to the Clavien–Dindo classification [22, 23]. We then reclassified the complication variables into “no complications” for cases with no complications or “having complications” for those with complications of grades I to IV.

Satisfaction with the decision to donate was measured using the following question: “If you go back to before your donation, would you still donate?” The response to this question was evaluated using a 4-point Likert scale: 1 = “definitely not,” 2 = “not likely,” 3 = “somewhat likely,” 4 = “very likely,” with a higher score reflecting higher satisfaction.

### Data analysis

Logistic regression was used to identify influential factors for poor PCS and MCS scores. Unadjusted and adjusted odds ratios and 95% confidence intervals (CI) were estimated using univariable and multivariable logistic regression models, respectively. Poor PCS and MCS scores, which were defined as scores more than 0.5 SD below the normative mean of the general Korean population [19], were coded as 1, and others were coded as 0. In the multivariable analysis, a predictor was unmet expectations for surgical outcomes, and possible confounding variables known to be associated with LLD’s PCS and MCS were controlled. These control variables were age, sex, education level, monthly income, postoperative complications, recipient death, time since donation, satisfaction with donation based on the previous research [6, 10–14]. The predictor and control variables were simultaneously entered into multivariable adjusted model.

In terms of unmet expectations for surgical outcomes, a dichotomous variable was generated by coding 0 when 0 to 2 items were rated as “worse than expected” and coding 1 when 3 or 4 items were rated as “worse than expected.” Among control variables, age, time since donation by year, and satisfaction with the decision were considered continuous variables. The other variables were dichotomous: sex (male “0”; female “1”), education level (less than bachelors’ degree “0”; bachelors’ degree or higher “1”), monthly income (less than 3.5 million Korean won, “0”; 3.5 million Korean won or more, “1”), postoperative complications (no complications, “0”; had complications, “1”), and recipient death (alive, “0”; death, “1”). The Hosmer–Lemeshow test was applied to evaluate the goodness-of-fit of the models. Missing data were noted for monthly income (2.4%), recipient death (1.6%), and SF-12v2 (0.7%), and the expectation-maximization algorithm was used to impute the missing values for SF-12v2. All statistical analyses were carried out using IBM SPSS Statistics (Version 26).

## Results

### Sample description

The demographic and donor-specific characteristics of LLDs are shown in Table 1. The mean age of the participants was 37.9 ± 11.4 years, ranging from 19 to 63 years. Among the 124 participants, 56.5% were male; 72.6% had a bachelor’s degree or higher; and 62.9% had a monthly income of less than 3.5 million Korean won. Majority of the LLDs were children of the recipient (71%). The mean of length of hospital stay was 9.5 ± 3.2 days.

**Table 1:** Characteristics of the living liver donors

N = 124	n (%)	Mean (SD)
Age at survey completion		37.9 (11.4)
19–29	37 (29.8)	
30–39	35 (28.2)	
40–49	29 (23.4)	
50–59	19 (15.3)	
60–63	4 (3.2)	
Sex		
Male	70 (56.5)	
Female	54 (43.5)	
Education level		
Less than a bachelor’s degree	34 (27.4)	
Bachelor’s degree or higher	90 (72.6)	
Monthly income (million KRW)		
< 3.5	78 (62.9)	
≥ 3.5	43 (34.7)	
Unknown	3 (2.4)	
Relationship to recipient		
Child	88 (71.0)	
Spouse	17 (13.7)	
Sibling	11 (8.9)	
Parent	8 (6.5)	
Length of hospital stay (days)		9.5 (3.2)
≤6	16 (12.9)	
≥7, ≤ 10	72 (58.1)	
≥ 11, ≤ 15	33 (26.6)	
≥16	3 (2.4)	
Clavien–Dindo classification		
None	64 (51.6)	
Grade I	50 (40.3)	
Grade II	7 (5.6)	
Grade III	3 (2.4)	
Recipient status		
Died	11 (8.9)	
Survived	111 (89.5)	
Unknown	2 (1.6)	
Time since donation (years)		2.1 (2.2)
< 1	34 (27.4)	
≥ 1, < 3	48 (38.7)	
≥ 3, <5	31 (25.0)	
≥ 5, < 10	9 (7.3)	
≥ 10	2 (1.6)	
Satisfaction with decision to donate		
Definitely not	1 (0.8)	
Not likely	3 (2.4)	
Somewhat likely	26 (21.0)	
Very likely	94 (75.8)	

Sixty LLDs (48.3%) experienced complications that were categorized as follows by the Clavien–Dindo classification: grade I, which included fluid collection, pleural effusion, subcutaneous emphysema, atelectasis, dizziness and nausea, keloid and hypertrophic scars, wound dehiscence, hematuria, vaginal oozing, fatty liver, chest pain, shoulder pain, fever, and temporarily elevated aspartate transaminase or/and alanine transaminase levels over at least 1 year after the transplantation; grade II, which included dyspepsia, gaseous distention, chronic cough, urticarial rash, diarrhea, gastroenteritis, colitis (all requiring antibiotics and etc.), and portal vein stenosis (requiring aspirin); grade IIIa, which included common bile duct stenosis, biloma, and pulmonary thromboembolism; or grade IIIb, which included hematoma.

A small proportion of LDLT recipients (8.9%) was deceased at the time point of survey completion. The time since donation ranged from 1 month to 12 years; two-thirds of the donors (66.1%) underwent surgery for transplant within 3 years. The majority of donors (75.8%) were very satisfied with their decision to donate their liver.

### Quality of life and unmet expectations about surgical outcomes

**Fig. 1:**
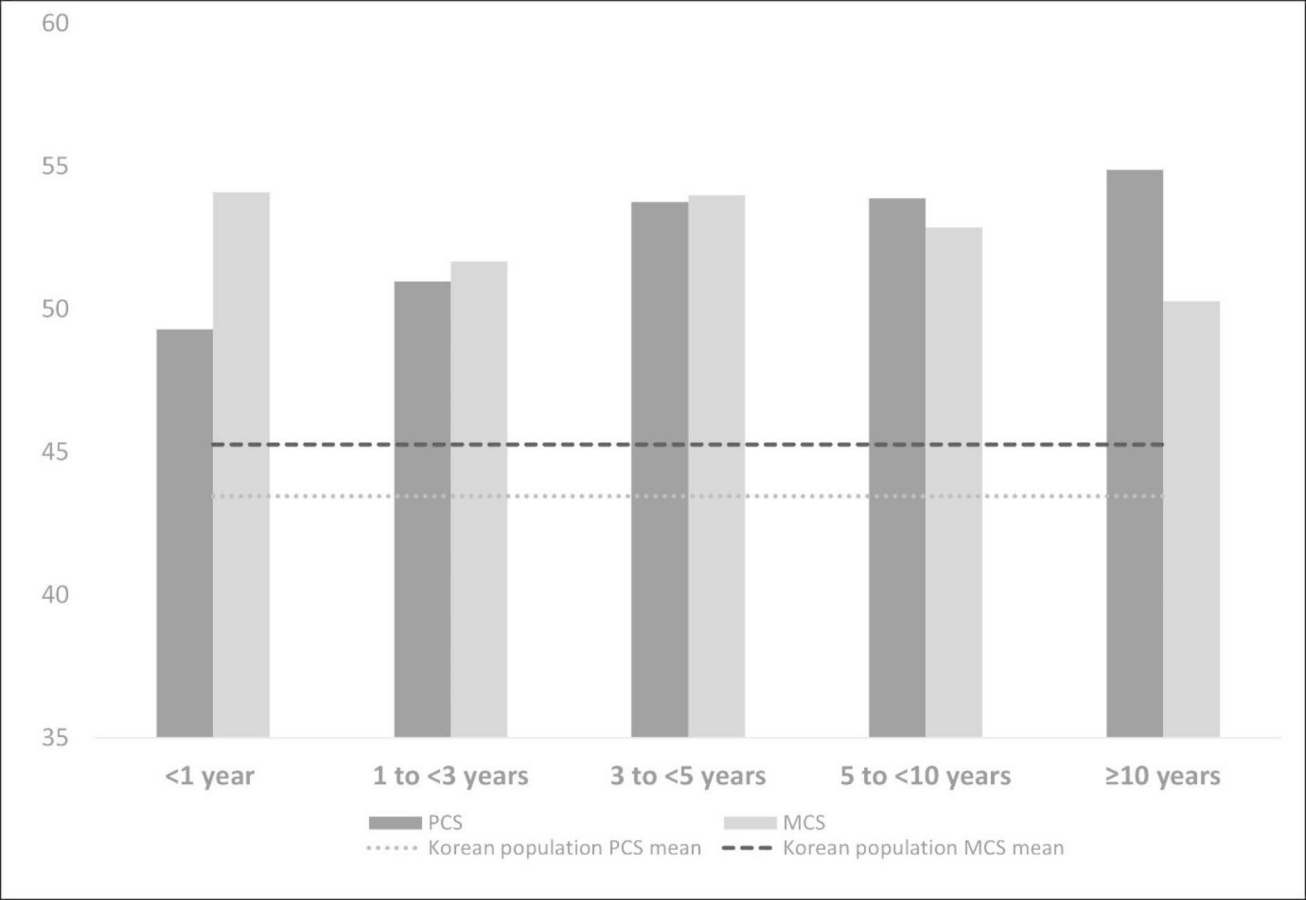
**Short Form-12 health survey (SF-12) component summary** The figure depicts the short Form-12 health survey (SF-12) component summary mean scores in LLD and general Korean population by time since donation

The mean donor SF-12 scores were 51.48 ± 7.44 (PCS) and 52.97 ± 8.47 (MCS) while the normative SF-12 scores for the general Korean population were 43.46 ± 3.05 (PCS) and 45.26 ± 4.35 (MCS) [19]. SF-12 component summary mean scores in LLD by time since donation and those of general Korean population are seen in Fig. 1. Among the study participants, 14 (11.3%) had poor PCS scores, and 16 (12.9%) had poor MCS scores. In terms of unmet expectations about surgical outcomes (Table 2), the percentages of the participants who reported worse-than-expected experiences for length of hospital stay, speed of recovery, pain, and complications were 9.7%, 22.6%, 34.7%, and 7.3%, respectively.

**Table 2:** Frequencies of the four items of postoperative experience in comparison with preoperative expectations

	Better than expected	As expected	Worse than expected (unmet expectations)
**Actual postoperative experience of**	n (%)	n (%)	n (%)
Length of hospital stay	69 (55.6)	43 (34.7)	12 (9.7)
Speed of recovery	60 (48.4)	36 (29.0)	28 (22.6)
Pain	54 (43.5)	27 (21.8)	43 (34.7)
Complications	98 (79.0)	17 (13.7)	9 (7.3)

### Logistic regression models

The final logistic regression model for PCS is shown in Table 3, and that for MCS is shown in Table 4. In the univariable model for PCS (Table 3), time since donation and unmet expectations for surgical outcomes were significantly associated with poor PCS scores (unadjusted odds ratio [UOR] 0.53, 95% CI 0.32–0.88; UOR 6.93, 95% CI 1.67–28.74). In the univariable model for MCS (Table 4), education level, satisfaction with decision to donate, and unmet expectation were statistically related to poor MCS scores (UOR 0.32, 95% CI 0.11–0.93; UOR 0.37, 95% CI 0.17–0.83; UOR 5.67, 95% CI 1.40–22.97).

**Table 3:** Logistic regression model for PCS scores (n = 124)

Variable	Unadjusted OR (95% CI)	p-value	Adjusted OR (95% CI)	p-value
Age	1.002 (0.954, 1.052)	0.940	1.013 (0.952, 1.078)	0.674
Female (ref: male)	1.855 (0.603, 5.710)	0.281	2.796 (0.681, 11.478)	0.154
Bachelor’s degree or higher (ref: less than a bachelor’s degree)	0.644 (0.199, 2.082)	0.463	0.890 (0.209, 3.799)	0.875
Monthly income: ≥ 3.5 million KRW (ref: <3.5 million KRW)	0.786 (0.227, 2.721)	0.704	0.626 (0.146, 2.677)	0.527
Postoperative complications (ref: no complications)	2.082 (0.656, 6.614)	0.214	1.351 (0.354, 5.166)	0.660
Recipient death (ref: recipient alive)	0.000 (0.000)	0.999	0.000 (0.000)	0.999
Time since donation (years)	0.529 (0.316, 0.884)	0.015	0.502 (0.265, 0.952)	0.035
Satisfaction with decision to donate	0.777 (0.311, 1.941)	0.589	0.859 (0.242, 3.047)	0.814
Unmet expectations for surgical outcomes: 3–4 items were worse than expected (ref: 0–2 items were worse than expected)	6.933 (1.673, 28.737)	0.008	7.461 (1.375, 40.488)	0.020

**Table 4:** Logistic regression model for MCS scores (n = 124)

Variable	Unadjusted OR (95% CI)	p-value	Adjusted OR (95% CI)	p-value
Age	1.003 (0.958, 1.050)	0.900	0.993 (0.936, 1.053)	0.809
Female (ref: male)	0.547 (0.178, 1.682)	0.293	0.351 (0.089, 1.389)	0.136
Bachelor’s degree or higher (ref: less than a bachelor’s degree)	0.317 (0.108, 0.929)	0.036	0.239 (0.060, 0.961)	0.044
Monthly income: ≥ 3.5 million KRW (ref: <3.5 million KRW)	0.895 (0.285, 2.810)	0.849	1.017 (0.241, 4.289)	0.982
Postoperative complications (ref: no complications)	1.077 (0.377, 3.078)	0.890	0.792 (0.220, 2.858)	0.722
Recipient death (ref: recipient alive)	0.640 (0.076, 5.366)	0.681	0.751 (0.071, 7.977)	0.812
Time since donation (years)	0.809 (0.583, 1.123)	0.205	0.889 (0.598, 1.322)	0.561
Satisfaction with decision to donate	0.373 (0.169, 0.826)	0.015	0.301 (0.117, 0.773)	0.013
Unmet expectations for surgical outcomes: 3–4 items were worse than expected (ref: 0–2 items were worse than expected)	5.667 (1.398, 22.966)	0.015	7.150 (1.346, 37.972)	0.021

For the multivariable logistic regression models for both PCS and MCS, age, sex, education level, monthly income, postoperative complications, recipient death, time since donation, and satisfaction with the decision to donate were controlled. The multivariable logistic regression model for PCS was significant (*X*^2^ (df = 9) = 19.313, *p* = .023) with acceptable goodness-of-fit statistics (Hosmer–Lemeshow: *p* = .177). In this adjusted model for PCS, unmet expectations for surgical outcomes predicted poor PCS scores (adjusted odds ratio [AOR] 7.46, 95% CI 1.38–40.49) after controlling for age, sex, education level, income, postoperative complications, recipient death, time since donation, and satisfaction with the decision to donate (Table 3). In other words, donors who reported three or four unmet expectations were more likely to have poor PCS scores than those who reported two or fewer unmet expectations. In this model, a shorter interval since donation was also associated with poor PCS scores (AOR 0.50, 95% CI 0.27–0.95).

The multivariable logistic regression model for MCS was significant (*X*^2^ (df = 9) = 18.638, *p* = .028) with acceptable goodness-of-fit statistics (Hosmer–Lemeshow: *p* = .266). In this adjusted model for MCS, unmet expectations for surgical outcomes were a predictor of poor MCS scores after controlling for other factors (AOR 7.15, 95% CI 1.35–37.97) (Table 4). The likelihood of poor MCS scores increased in donors who had three or more items of unmet expectations than in those who had two or fewer items of unmet expectations (AOR 7.15, 95% CI 1.35–37.97). In addition, LLDs having less than a bachelor’s degree and less satisfied with decision to donate were likely to experience poor MCS (AOR 0.24, 95% CI 0.06–0.96; AOR 0.30, 95% CI 0.12–0.77).

## Discussion

This study explored the quality of life of LLDs in terms of its relationship with the donors’ unmet expectations regarding surgical outcomes. Experiencing a worse-than-expected recovery process was associated with poorer physical and mental quality of life, even after controlling for age, sex, education level, income, presence of complications, recipient death, time since donation, and satisfaction with the decision to donate.

This study examined the unmet expectations of LLDs related to surgical outcomes by comparing preoperative expectations and actual experiences. The surgical outcome that most frequently showed a discrepancy between expectation and reality was pain (34.7%), while the outcome where such discrepancies were least frequently reported was complication (7.3%). Meanwhile, 8.1% of LLDs answered that three or four surgical outcomes were worse than anticipated. They were more likely to have lower mental and physical HRQOL than those who reported unfulfilled expectations regarding two or fewer surgical outcomes. Unmet expectations about surgical outcomes such as the length of hospital stay, speed of recovery, pain, and complications can increase psychological distress and symptom-related discomfort after surgery [15]. In the current study, the unmet expectations of LLDs were associated with poorer mental and physical HRQOL.

All patients’ expectations regarding surgical outcomes cannot be exactly the same as the actual postoperative experience, and unpredictable outcomes and uncertainty may still exist [20, 24]. Moreover, it is common for patients to have unrealistic optimism and expect unreasonably good surgical outcomes [15]. To decrease these discrepancies between expectations and reality, efforts must be made to help LLDs realistically anticipate surgical outcomes. The healthcare institution where this study was conducted tries to provide sufficient and adequate information to LLDs. In this study, the items in which proportion of unmet expectation was less than 10% were length of hospital stay and complications. The mean hospital stay was similar to the information provided through counseling before donation, and possible complications were elaborated in the informed consent document as well as the counseling content material (see Additional file 1). However, in these documents, information with regard to speed of recovery and pain was relatively deficient and vague. The information given to LLDs needs to include an explanation of the uncertain factors as well as certain factors related to surgical outcomes [24]. Although LLDs have reported that they were given appropriate and sufficient information [25], the information needs to be examined to determine if it is extensive, accurate, and actual and supports LLDs in maintaining realistic expectations. In addition, Weng et al. [26], who conducted a qualitative study of Taiwanese LLDs, showed that they may not really receive information about negative surgical outcomes to reduce their anxiety about donation. Therefore, a careful approach regarding LLDs’ attitudes toward possible negative surgical outcomes needs to be taken, and research to explore its impact on their unrealistic optimism should be performed.

In assessments of the HRQOL of LLDs, the mean donor SF-12 scores were 51.48 ± 7.44 (PCS) and 52.97 ± 8.47 (MCS), which appear to be higher than the normative SF-12 scores for the general Korean population: 43.46 ± 3.05 (PCS), 45.26 ± 4.35 (MCS) [19]. Because a liver donor is expected to have passed a medical evaluation, including general physical and mental health examinations, to become a donor [27], such evaluations may select individuals healthier than the general population. Moreover, the age of the general population ranged from 20 to 75 years [19], whereas that of this study sample ranged from 19 to 63 years.

In this study, a shorter period since donation was significantly associated with poor PCS scores. This result is consistent with that of prior studies in which participants reported their HRQOL within one or three postoperative years [13,28]. Furthermore, poor physical well-being is particularly associated with an interval of less than three months since donation [12, 29]. However, our outcome is in contrast to that reported by Ladner et al. [12], who suggested that a longer interval from donation increased the likelihood of poor PCS scores. This may also be a plausible result since the postoperative period in their study was relatively evenly distributed from one to eight years and the study participants had been aging in a longitudinal study, which was performed for 11 years.

In our study population, 96.8% of LLDs were satisfied with the decision to donate; thus, 3.2% of LLDs had decisional regrets about liver donation. We found that the more satisfied the donor was with the decision to donate, the more likely he or she was to have better mental HRQOL. This is consistent with the findings of a study on kidney donors by Wirken et al. [14], which suggested that donors with regret experienced poorer HRQOL, especially in the social functioning and health perception domains.

This study had some limitations. First, to measure LLDs’ HRQOL, we used a generic instrument, the SF-12, instead of a donor-specific instrument. This could have disregarded some distinct aspects of the donors; nevertheless, SF-12 is highly utilized and thus suitable for comparison with various populations from different countries. Moreover, unmet expectations were measured based on retrospective questions, leading to the possibility of recall bias. Furthermore, a prospective longitudinal study may have to be performed to compare preoperative expectations and actual postoperative experiences. Finally, our results were drawn from a survey at one transplantation center at a university hospital; thus, the results had limited generalizability.

## Conclusion

This study explored LLDs’ unmet expectations related to surgical outcomes. Healthy LLDs decide to become surgical patients for altruistic reasons and most are satisfied with their decision to donate during the rest of their lives. However, they go through the postoperative recovery process, and unmet expectations regarding surgical outcomes during this process may have a negative effect on donors’ HRQOL. Thus, we need to support LLDs in obtaining more accurate information to decrease unmet expectations.

## Electronic supplementary material

Below is the link to the electronic supplementary material.

Supplementary Material 1

## Data Availability

The dataset used and analyzed during the current study is available from the corresponding author on reasonable request.

## Abbreviations

AOR: adjusted odds ratio.
BP: Bodily pain.
CI: Confidence intervals.
GH: General health.
HRQOL: Health-related quality of life.
LDLT: Living-donor liver transplantation.
LLD: Living liver donor.
MCS: Mental component summary.
MH: Mental health.
PCS: Physical component summary.
PF: Physical functioning.
RE: Role emotional.
RP: Role physical.
SF: Social functioning.
UOR: unadjusted odds ratio.
VT: Vitality.
