# Supplementary material for: Does living liver donors’ underestimation about surgical outcomes impact on their health-related quality of life after donation?: a descriptive cross-sectional study
Source: Health Qual Life Outcomes. 2022 Oct 24;20:146. doi: 10.1186/s12955-022-02055-0 (PMC9594971; doi:10.1186/s12955-022-02055-0)
Supplement: Supplementary file 1 — Supplementary Material 1 [file 12955_2022_2055_MOESM1_ESM.docx]

**Does living liver donors’ underestimation about surgical outcomes impact on their health- related quality of life after donation?**

**Supplementary material 1. Information provided prior to surgery by the transplantation center regarding the length of hospital stay, speed of recovery, pain, and complications**

**I. Some excerpts from LDLT counseling content material before donation from the liver transplant center**

5. Liver donor’s hospitalization & recovery period

After surgery, LLD is moved to a recovery room, and after awakening from anesthesia, they are transferred to a surgical ward. One can be discharged from the hospital approximately 5–7 days after surgery, followed by visits to an outpatient clinic after a week, then a month, four months, and an year to check the liver function through blood tests and imaging examinations (e.g., CT scan). After this, it is also advisable to maintain regular annual follow-ups on one’s health at the outpatient clinic.

After discharge from the hospital, the patient needs to be stable and have adequate rest and nutrition for recovery. They are supposed to refrain from strenuous muscle exercises, long drives, and physical work. During recovery, one should adhere to abstinence from alcohol and smoking. If the patient does not experience any complications, their daily activities and social life will return to normal.

8. Postoperative complications and side-effects

During surgery, the donor’s gall bladder is extracted, and complications related to general anesthesia or hepatectomy can occur (e.g., surgical scar, wound complications, bleeding, pain, infection, pneumonia, intestinal adhesion, bile leak, intra-abdominal abscess, biliary stricture, cholangitis, and liver failure). The incidence of major complications is approximately 2% and may require additional treatment. Global reports indicate that the mortality rate of liver donors is 0.05-0.1%, and there have been no cases of death in LLDs at this transplant center.

10. Surgical wound

| Open surgery | Pure laparoscopic surgery |
| --- | --- |
| 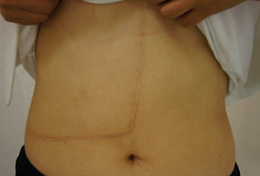 | 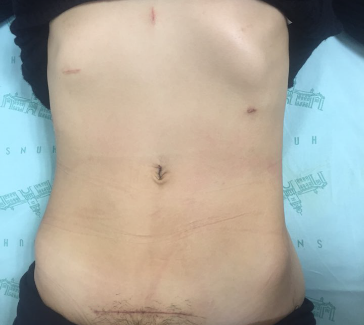 |

● Surgical wound size varies depending upon the individual’s skin status and postoperative wound management.

● During surgery, it is possible that the patient status determines the conversion from performing pure laparoscopic surgery to laparotomy.

II. **Some excerpts from an informed consent document for hepatectomy**

6. Potential complications/sequela/side effects and proposed management in case of the problems

1) Cardiovascular complications: arrhythmia, acute myocardial infarction, cerebral hemorrhage, cerebral infarction, and others.

This surgery involves significant hemodynamic changes during the surgical procedure, and even if the patient does not have any underlying cardiovascular diseases, they may experience severe or minor cardiovascular complications during or after surgery. This rarely leads to death.

2) Bleeding

The liver generates hemostatic substances, and severe liver cirrhosis decreases the number of hemostatic substances and platelets. Liver surgery always carries the risk of injuries to major vessels close to the liver, such as the hepatic portal vein and inferior vena cava. Occasionally, massive bleeding might occur which can be life-threatening. Therefore, a large amount of transfusion may be required, and this could be followed by complications such as electrolyte imbalance, infection, and hypothermia.

3) Thrombosis

To prevent thrombus formation in major vessels such as the hepatic artery or hepatic portal vein, antithrombotic treatment may be required during the perioperative period. Severe thrombosis in major vessels can occur after surgery and may require reoperation or imaging intervention.

4) Infection, bile leak, intra-abdominal abscess

Liver resection creates a large space filled with body fluids or bile leakage from the liver and the biliary system. In most cases, bile leakage is treated naturally by being obstructed, but sometimes it may require endoscopic or radiological biliary drainage procedures or surgical management.

Such a collection of body fluids, blood, and bile could get infected and develop into an abscess, causing fever and the stomachache. This may require antibiotics or an intervention using the radiological drainage procedure.

5) Biliary stricture, cholangitis, vascular-related complications

While rare, stricture of blood vessels flowing into or out of the biliary tract or liver can occur. Pertaining to the stricture, postoperative cholangitis could possibly recur. Most cases can be treated with medications, such as antibiotics or antithrombotic agents; some cases deteriorate into liver dysfunction that may require endoscopic/imaging intervention or additional surgery, and some into a fatal infection requiring further management.

6) Liver failure

If the remnant of the liver cannot recover its function spontaneously, liver function gradually worsens, and the following problems can occur in some cases: a disorder of nutrition supply and storage, jaundice and prolonged bile retention due to biliary excretion disorder, bleeding from exacerbation of hemostatic function, secondary deterioration of kidney function, systemic organ failure, and death.

7) Surrounding organ damage

Not only can vascular injuries occur, but the duodenum, spleen, kidney, colon, and the bile duct could also be damaged, or their function could decline. Such injuries and hypofunction may require further treatment.

8) Ascites

After liver resection, ascites occurs in 80% of patients with liver cirrhosis. If necessary, diuretics will be used; if it progresses rapidly, abdominal paracentesis may be considered.

9) Surgical wound complications (poor wound healing)

If the wound becomes infected, repeated dressing changes are performed. Surgical treatment is required for severe cases.

10) Anesthetic complications

Side-effects from general anesthesia

11) Respiratory complications

During surgery, the lung could partially collapse or form phlegm. In particular, during liver surgery, sometimes the right diaphragm is largely poked, and excess fluid is collected in the lungs, leading to the risk of atelectasis and pneumonia. One should try to take a deep breath, produce sputum, and pat their back. If fluid accumulation in the lung leads to high fever or shortness of breath, chest tube insertion is needed to drain the fluid or transfer to an intensive care unit may be needed for treatment.

12) Pain

Patient-controlled analgesia (PCA) is used after surgery. After a few days later, the pain gradually subsides. However, at the end of PCA injection, one can feel more pain. If intolerable pain is experienced even after several days, various tests and examinations are needed to check for complications.

13) Intestinal adhesion

The intestine and surrounding structures can adhere to each other, and intestinal obstruction requiring surgery can occur even a few years after the surgery.

7. Possibility of mortality related to the surgery (brain death or cardiac death)

Because of preoperative health status and many different reasons directly or indirectly to the surgery, it could lead to cardiac or brain death. Worldwide reports indicate that it can occur in 1% of all patients undergoing hepatectomy.

8. Things to be observed by the patients before and after surgery

2) After surgery

Immediately after surgery, complications such as bleeding from liver resection may occur; therefore, absolute rest is required. From the day after surgery, breathing exercises and ambulation should be started gradually. About a week after surgery, wound sutures are removed, and it takes approximately 3 months or more for the surgical wound to fully heal. Until this time, the pain may persist.

3) Recovery process

Postoperative patient condition determines transfers to an intensive care unit or to a ward with various injection tubes, drainage tubes, urine catheters placed, for transfusion and assessment of patient status. As the patient’s condition improves, the tubes are removed individually.

Typically, when passing gas is possible again, meals can be started, which may be changeable depending on the condition. Approximately a week after the surgery, total stitch-out is performed at the incision sites.
